# The Regulatory Effects of Interleukin-4 Receptor Signaling on Neutrophils in Type 2 Immune Responses

**DOI:** 10.3389/fimmu.2019.02507

**Published:** 2019-10-24

**Authors:** Cecilie Egholm, Lukas E. M. Heeb, Daniela Impellizzieri, Onur Boyman

**Affiliations:** ^1^Department of Immunology, University Hospital Zurich, Zurich, Switzerland; ^2^Faculty of Medicine, University of Zurich, Zurich, Switzerland

**Keywords:** neutrophil, type 2 immunity, interleukin-4, interleukin-13, interleukin-4 receptor, inflammation, helminth, neutropenia

## Abstract

Interleukin-4 (IL-4) receptor (IL-4R) signaling plays a pivotal role in type 2 immune responses. Type 2 immunity ensures several host-protective processes such as defense against helminth parasites and wound repair, however, type 2 immune responses also drive the pathogenesis of allergic diseases. Neutrophil granulocytes (neutrophils) have not traditionally been considered a part of type 2 immunity. While neutrophils might be beneficial in initiating a type 2 immune response, their involvement and activation is rather unwanted at later stages. This is evidenced by examples of type 2 immune responses where increased neutrophil responses are able to enhance immunity, however, at the cost of increased tissue damage. Recent studies have linked the type 2 cytokines IL-4 and IL-13 and their signaling via type I and type II IL-4Rs on neutrophils to inhibition of several neutrophil effector functions. This mechanism directly curtails neutrophil chemotaxis toward potent intermediary chemoattractants, inhibits the formation of neutrophil extracellular traps, and antagonizes the effects of granulocyte colony-stimulating factor on neutrophils. These effects are observed in both mouse and human neutrophils. Thus, we propose for type 2 immune responses that neutrophils are, as in other immune responses, the first non-resident cells to arrive at a site of inflammation or infection, thereby guiding and attracting other innate and adaptive immune cells; however, as soon as the type 2 cytokines IL-4 and IL-13 predominate, neutrophil recruitment, chemotaxis, and effector functions are rapidly shut off by IL-4/IL-13-mediated IL-4R signaling in neutrophils to prevent them from damaging healthy tissues. Insight into this neutrophil checkpoint pathway will help understand regulation of neutrophilic type 2 inflammation and guide the design of targeted therapeutic approaches for modulating neutrophils during inflammation and neutropenia.

## Introduction

Neutrophil granulocytes (neutrophils) are the most abundant leukocytes in human blood accounting for ~60–70% of immune cells in circulation at steady state (1). With a very short life span of approximately 5 days, there is a constant need for replenishment of this vast pool (2). Neutrophils are generated in the bone marrow at a rate of 1–2 × 10^11^ cells per day (3). Their release from the bone marrow is regulated by the signaling of C-X-C chemokine receptors (CXCR) 2 and 4. CXCR2-binding chemokines, CXCL8 in humans and CXCL1 and CXCL2 in mice, promote the mobilization of neutrophils and their egress into the blood stream (4, 5). Conversely, the engagement of CXCR4 with its ligand CXCL12 presented on bone marrow stromal cells keeps the newly-formed granulocytes in their bone marrow niche (6). At steady state, this delicate interplay ensures the maintenance of a stable peripheral blood pool of neutrophils. In case of inflammation or infection, pro-inflammatory mediators, such as granulocyte colony-stimulating factor (G-CSF), are produced by the affected tissue and shift the balance toward increased neutrophil generation and mobilization, for example by increasing CXCR2 and decreasing CXCR4 surface expression on neutrophils (7, 8).

**Figure 1:**
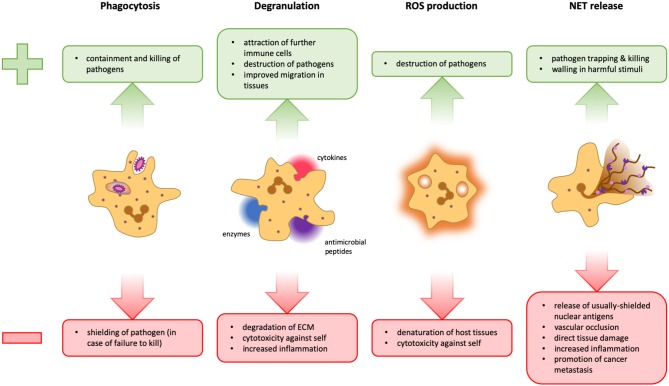
Advantages and disadvantages of neutrophil effector functions. During phagocytosis, microbes are engulfed and degraded in specialized organelles called phagolysosomes. This is a very powerful and clean method to dispose of pathogens because it takes place in a contained space, thus preventing widespread inflammation. However, should a pathogen manage to survive intracellular degradation, it is protected from extracellular factors and other immune cells that could potentially contain it **(Left)**. Neutrophils can release several different cytokines, antimicrobial peptides, and granular enzymes into their surroundings, a response termed degranulation. This mechanism facilitates migration within the tissue, activates and attracts other immune cells and can help fight pathogens that cannot be phagocytosed. However, the release of too many cytokines can lead to an overshooting immune activation. Tissue degradation could aid the spreading of pathogens and destroy the structural basis of organs, and several granular proteins are also toxic for host cells **(Middle Left)**. Neutrophils can release reactive oxygen species (ROS) into phagolysosomes as well as into the extracellular space. These molecules can potentiate pathogen killing, but are not selective and can therefore also damage host cells **(Middle Right)**. The formation of NETs can be an efficient means to trap and kill pathogens and potentially walling off harmful stimuli. However, the release of cytoplasmic and nuclear proteins can cause the formation of autoantibodies, favoring autoimmunity. Furthermore, NETs can obstruct blood vessels and glandular ducts leading to inflammation. Granular proteins attached to the chromatin fibrils damage host tissue and the release of pro-inflammatory mediators may result in overshooting inflammation. Moreover, NETs have also been implicated in the facilitation of tumor metastasis **(Right)**.

Neutrophils are typically the first non-resident immune cells that arrive at a site of inflammation (9, 10). Upon local activation of the vasculature, endothelial cells present chemokines and cell adhesion molecules at their luminal side (11). These ligands are recognized by their counterparts on the surface of bypassing neutrophils, leading to deceleration and eventually firm adhesion of the leukocytes to the endothelium. The neutrophils then crawl along the vessel wall following fixed gradients of so-called intermediary chemoattractants, particularly CXCR2-binding chemokines, before they transmigrate through the endothelium into the interstitial space. There, they advance to their destination by tracking gradients of other chemotactic stimuli such as N-formylmethionine-leucyl-phenylalanine (fMLP) released by bacteria (12, 13). These end-target chemoattractants override the signals emanating from intermediary chemokines (14). Once they reach the site of infection, neutrophils employ various mechanisms to kill or inactivate pathogens, including phagocytosis, degranulation, production of reactive oxygen species (ROS), and neutrophils extracellular trap (NET) formation (Figure 1; discussed in section Neutrophil Effector Functions).

Given the abundance and readiness of these heavily-armed immune cells, overshooting neutrophil activity can lead to detrimental tissue damage. Thus, mechanisms need to be in place, which keep neutrophil responses in check but at the same time allow efficient clearance of pathogens. Although neutrophils have traditionally been associated with type 1 and type 3 immunity, they have recently been found to contribute to type 2 immune responses, thus also aiding in the removal of helminth parasites (15, 16). Conversely, neutrophils are often conspicuously absent from tissues where the type 2 cytokines interleukin (IL)-4 and IL-13 dominate (17, 18). In this review, we will propose a mechanism that connects these seemingly contradictory findings. We will first summarize beneficial and harmful effects of neutrophils in type 1, type 2, and type 3 immune responses. We will then focus on a growing body of evidence that suggests involvement of neutrophils in type 2 immunity. Finally, we will discuss recent results from mouse and human neutrophil research that have demonstrated how IL-4 and IL-13 receptor signaling inhibits several neutrophil effector functions (19–21), and we will elaborate on a temporal model unifying the reported findings.

## Neutrophil Effector Functions

### Phagocytosis

The most prominent neutrophil effector function is phagocytosis, the process during which pathogens are recognized, encapsulated and internalized in membranous vesicles, and digested by fusion of the vesicles with lysosomes and granules containing enzymes and antimicrobial peptides. The recognition step usually depends on pathogen- and damage-associated molecular patterns (PAMPs and DAMPs, respectively) as well as opsonization of the pathogen by antibodies or complement factors that bind to Fc or complement receptors on the neutrophil, respectively (22–24). Engagement of these receptors leads to the active engulfment of the pathogen. Subsequently, lysosomal hydrolases and granular enzymes are released into the vesicle by fusion with the respective organelles and start the destruction of the phagocytosed material (25). Additionally, the nicotinamide adenine dinucleotide phosphate (NADPH) oxidase complex is assembled on the phagolysosome membrane, leading to the formation and discharge of superoxide anions (${\text{O}}_{2}^{-}$) into the lumen. These harm pathogens directly or react further into other ROS, all of which have antimicrobial activity (see below) (26).

### Degranulation

Neutrophils possess a large pool of intracellular vesicles (granules) whose contents can be released into the extracellular space or into phagosomes in a process called degranulation. Not all neutrophil granules are the same: There are four very distinct types of secretory organelles that can be distinguished by their content, membrane proteins, function, and time point of mobilization. Primary or azurophilic granules contain the most potent cargo: myeloperoxidase (MPO), defensins, neutrophil elastase (NE), and cathepsins. These proteins are powerful antimicrobial agents and are released only after several checkpoints have been passed and the neutrophil is in closest proximity to the pathogen. Secondary or specific granules also accommodate antimicrobial factors (e.g., lactoferrin, neutrophil-gelatinase associated lipocalin) together with matrix metalloproteases (MMPs) neutrophil collagenase (MMP-8) and leukolysin (MMP-25). The antimicrobial factors provide protection against pathogens while the MMPs can degrade extracellular matrix and thus enable more efficient neutrophil migration through tissues. Tertiary or gelatinase granules predominantly contain their namesake gelatinase (MMP-9) which also facilitates migration. The fourth type are secretory vesicles, which serve as reservoirs of membrane proteins that are needed on the cell surface upon priming, such as the CD11b–CD18 heterodimer (α_M_β_2_-integrin, Mac-1, complement receptor 3), CD35 (complement receptor 1), and the fMLP receptor. They do not contain notable soluble cargo. Besides this multitude of proteins working *in situ*, neutrophils are also capable of releasing several chemokines, cytokines, and growth factors that recruit other immune cells to a site of inflammation (27–32).

Despite being an essential means of combating infection, degranulation can also cause serious damage if not kept in check. While MMPs may be beneficial for cell migration, they can also destroy the connective tissue, and several constituents of the azurophilic granules not only kill pathogens, but are also cytotoxic to host cells (33) (Figure 1). Moreover, uncontrolled recruitment and activation of immune cells leads to excessive inflammation. It is thus not surprising that over-shooting degranulation of neutrophils has been associated with several inflammatory disorders such as septic shock, severe lung injury, rheumatoid arthritis, and severe asthma (34, 35).

### Reactive Oxygen Species

ROS are a group of small, unstable oxygen-based molecules. Owing to their chemical instability, they are highly reactive and can denature or damage proteins, lipids, and DNA (36). In neutrophils, ROS are produced in a process called respiratory or oxidative burst by the multi-unit enzyme complex NADPH oxidase (37). The different subunits of the enzyme are stored in separate compartments of the cell to prevent accidental generation of ROS. Upon activation, the NADPH oxidase assembles on the phagosomal or plasma membrane and produces and releases superoxide anions into the phagosome or extracellular space, respectively (38). Superoxide can react further with protons to hydrogen peroxide (H_2_O_2_), which is used by MPO to create hypochlorous acid (HClO) (39). All these types of ROS have microbicidal activity, but also consume protons in their chain of reactions, thereby neutralizing the acidic content from lysosomes and granules (40). This in turn facilitates the liberation of NE and cathepsins, which at low pH are tightly bound to proteoglycans and thus less active (41). In fact, ROS likely participate less in direct pathogen killing, but they rather facilitate proper activation of azurophilic granule enzymes, which cause pathogen killing in the phagosome (42).

Whether ROS are directly or indirectly responsible for pathogen killing, they are a vital part of innate immunity. In fact, bacterial strains that are able to disarm ROS by producing superoxide dismutase (SOD), which catalyzes the reaction of ${\text{O}}_{2}^{-}$ into O_2_ and H_2_O_2_, and catalase, which in turn catalyzes the decomposition of H_2_O_2_ into O_2_ and H_2_O, are much more virulent than their SOD- or catalase-negative counterparts (43). Another example is chronic granulomatous disease (CGD), a genetic disorder affecting the NADPH oxidase, which renders patients incapable of producing ROS. These patients suffer from frequent and recurrent infections, also with opportunistic pathogens (44). Despite their importance in combatting infection, unchecked production of extracellular ROS leads to tissue damage by virtue of their lack of pathogen specificity (45) (Figure 1).

### Neutrophil Extracellular Traps

NETs are meshes of DNA decorated with antimicrobial peptides that can be released by neutrophils in response to various stimuli. They were first described by Brinkmann et al. as a novel mechanism of how neutrophils can combat infection (46). Pathogens, mainly yeast and bacteria, stick to the DNA fibrils, which prevents them from spreading in the tissue, and they are degraded by the granule proteins that are attached to the chromatin network (47). Since 2004, numerous stimuli have been described to induce NET formation, of which large pathogens seem to be the main trigger (48). The exact process of how NETs form remains an active area of research. The most widely accepted model involves chromatin decondensation including histone citrullination, disintegration of nuclear, and granule membranes, intracellular mixing of the components and, finally, release into the extracellular space (49). Some reports provided evidence that the NADPH oxidase was necessary for NET formation. Interestingly, however, despite their lack of a functional NADPH oxidase CGD patients have been shown to form NETs by using mitochondrial ROS (50). It seems that depending on signal type and strength, NET formation can be fast and non-lytic, leaving behind an intact anuclear cytoplast (51), or slow and lytic, spilling the cell contents while the NET breaks free from the cell membrane (52). Some studies also present the possibility of NET release by living cells using mitochondrial in lieu of nuclear DNA (50, 53).

NETs have been shown to have several beneficial properties. Their main use is in immobilizing and degrading bacteria, fungi, and viruses (54–56). Another less prominent function is the shielding of damaged tissues that might otherwise elicit an unwanted inflammation (57). As helpful these mechanisms may be, NETs have also been implicated as players in a multitude of different diseases. Firstly, the release of nuclear material into the extracellular space provides access to otherwise shielded antigens and may result in the formation of autoantibodies (Figure 1), as suggested for rheumatoid arthritis (RA), systemic lupus erythematodes, anti-phospholipid syndrome, and anti-neutrophil cytoplasmic antibody (ANCA)-associated vasculitis (58–61). Secondly, the massive release of proteins can be a double threat. On the one hand, cytokines may drive inflammation leading to tissue damage or atherosclerosis (62). On the other hand, the proteases associated with NETs may degrade cytokines and chemokines, resulting in a possibly unwanted anti-inflammatory effect (63). Moreover, the giant and sticky structures of NETs can occlude blood vessels, leading to thrombosis or sepsis (14). Finally, NETs have also been proposed as players in cancer dissemination and metastasis formation (64, 65) (Figure 1).

## Role of Neutrophils in Different Types of Immune Responses

**Figure 2:**
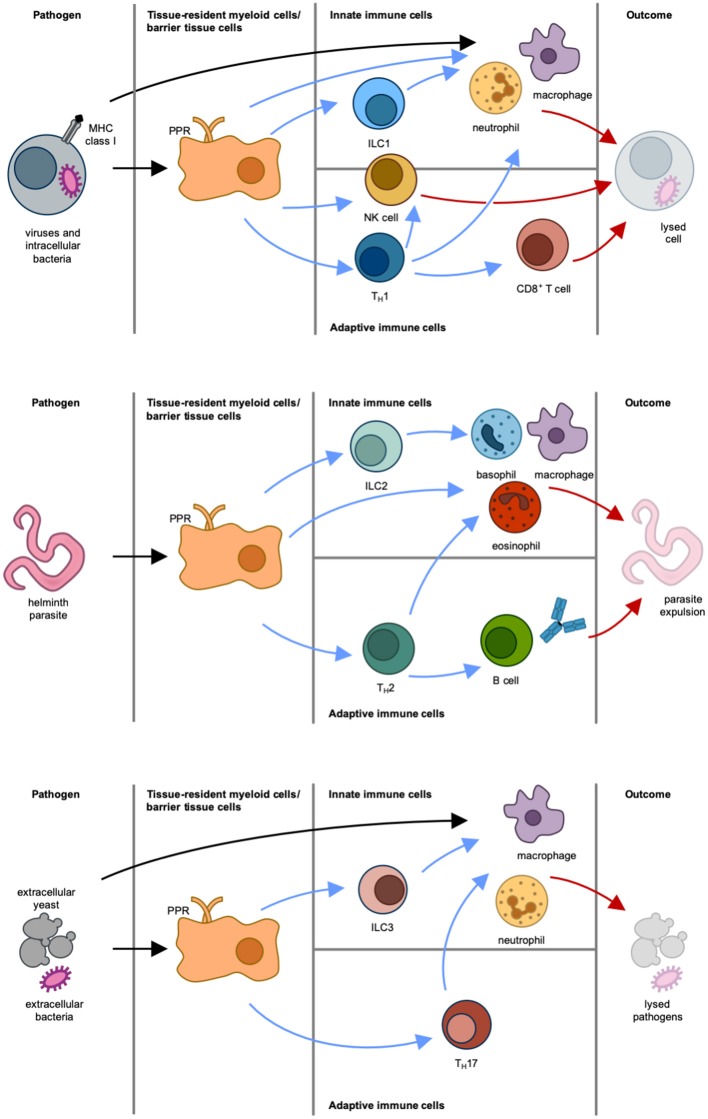
Different types of immune responses. Type 1 immune response **(Top):** Intracellular pathogens induce differentiation of naïve T cells into T_H_1 cells via antigen-presenting cells and IL-12. IFNγ is the main effector cytokine that stimulates and actives NK cells, type 1 innate lymphoid cells (ILC1), and T_H_1 cells, which in turn activate CD8^+^ cytotoxic T cells, macrophages and neutrophils. NK cells, macrophages and neutrophils can also be directly activated via pattern recognition receptor (PRR)-mediated recognition of pathogen-associated molecular patterns (PAMPs). The result of this immune response is lysis of cell and pathogen. Type 2 immune response **(Middle):** Parasites induce epithelial damage, which leads to the release of thymic stromal lymphopoietin (TSLP), IL-25, and IL-33 from epithelial cells. This in turn causes both differentiation of naïve T cells into T_H_2 cells via antigen-presenting cells and stimulation of ILC2, mast cells, basophils and eosinophils. In response to this, the activated immune cells produce IL-4, IL-5, IL-9, and IL-13. These effector cytokines stimulate B cells and induce isotype switching to immunoglobulin E and differentiation of macrophages to alternatively-activated macrophages (also termed M2 macrophages). The result of this immune response is parasite expulsion. Type 3 immune response **(Bottom)**: Extracellular pathogens induce differentiation of naïve T cells into T_H_17 cells via antigen-presenting cells and IL-23. Production of IL-17 and IL-22 by ILC3 and T_H_17 cells leads to the activation of macrophages and neutrophils. The result of this immune response is pathogen killing. Initiation of immune response is indicated by black arrows. Blue arrows mark stimulatory signals by cytokines produced by immune cells. Effector responses are indicated by red arrows.

The immune system has evolved different types of effector immune responses to counter the various pathogens. These are commonly referred to as type 1, type 2, and type 3 immunity, and each engage different subtypes of innate lymphoid cells (ILCs) and other innate immune cells, CD4^+^ helper T (T_H_) and CD8^+^ cytotoxic T cells, as well as CD4^+^ follicular helper T (T_FH_) cells and antibody responses by B cells, as discussed below (Figure 2).

### Type 1 Immunity

Type 1 immunity serves to protect against intracellular pathogens by the production of the effector cytokine interferon-γ (IFNγ) and engagement of type 1 ILCs (ILC1), natural killer (NK) cells, type 1 T_H_ (T_H_1) cells, CD8^+^ cytotoxic T cells, type 1 T_FH_ cells and immunoglobulin (Ig) G1 and IgG3 antibody responses. Together with these immune cells, also macrophages and neutrophils play a central role in type 1 immunity. These innate cells can directly detect foreign microbes via PAMPs, which they recognize by pattern recognition receptors (PRRs) (66). During infection with the intracellular pathogen *Listeria monocytogenes*, neutrophils were shown to have an important protective role. This was particularly seen in the liver, which is one of the primary target organs of this bacteria. Here, the early phagocytosis mediated by recruited neutrophils was essential for limiting bacterial spread and controlling infection (67–69). It has also been suggested that IFNγ has a direct effect on neutrophil activation and can induce MHC class II expression, at least *ex vivo* (70). Although macrophages have a protective role against intracellular pathogens, they are also very susceptible to becoming infected themselves. In the case of *Mycobacterium tuberculosis* infection of macrophages, it has been reported that neutrophil granules can be transferred to macrophages and facilitate the clearance of chronically infected cells (71).

### Type 2 Immunity

**Figure 3:**
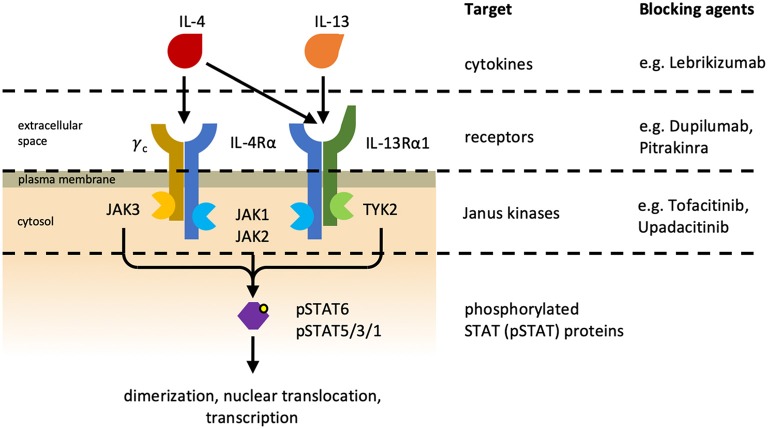
Interleukin-4 receptors. Different heterodimeric IL-4 receptors (IL-4Rs) exist and they share the IL-4Rα subunit. The type I IL-4R **(Left)** consists of IL-4Rα and the common gamma chain cytokine receptor (γ_C_), and the type II IL-4R **(Right)** is made of IL-4Rα and IL-13Rα1. IL-4 can associate with and signal via both IL-4Rs, whereas IL-13 can only bind to and signal via the type II IL-4R. Additionally, IL-13 can bind to IL-13Rα2 (not depicted), which is referred to as non-signaling decoy receptor. Cytokine-mediated receptor dimerization leads to the activation of receptor-associated Janus kinases (JAK) and consequently to the phosphorylation and activation of signal transducer and activator of transcription (STAT) proteins. Phosphorylated STAT (pSTAT) proteins subsequently dimerize and translocate to the nucleus where they initiate transcription of their target genes. Different treatment strategies have been developed to inhibit IL-4R signaling. Lebrikizumab is a neutralizing anti-IL-13 antibody. Pitrakinra, an IL-4 antagonist, and Dupilumab, an IL-4Rα-blocking antibody, are both preventing cytokine–receptor interaction. JAKs can also be inhibited pharmaceutically by small molecules. Upadacitinib is a selective JAK1 inhibitor currently being investigated in clinical trials, whereas Tofacitinib inhibits both JAK1 and JAK3 and is approved for the treatment of rheumatoid arthritis and psoriatic arthritis (76).

Type 2 immunity has evolved to efficiently induce resistance and tolerance to parasitic infections, especially helminthic infestations. Type 2 immunity is typically initiated by the activation of epithelial cells and PRR-expressing myeloid cells that secrete IL-25, IL-33, and thymic stromal lymphopoietin (TSLP). In response to these cytokines, type 2 ILCs (ILC2) begin to produce IL-5 and IL-13, which induce the differentiation of CD4^+^ T cells to type 2 T_H_ (T_H_2) cells, which in turn secrete the characteristic type 2 cytokines IL-4, IL-5, IL-9, and IL-13 (72–74). This cytokine milieu fosters the development and proliferation of other cells involved in type 2 immunity, including basophils, eosinophils, mast cells, and NKT cells, and drives the differentiation of type 2 T_FH_ cells and IgE antibody responses. Especially IL-4 and IL-13 are central cytokines in type 2 immunity, and they both signal via the IL-4 receptor (IL-4R) system (75). Two types of IL-4Rs exist (Figure 3): type I IL-4Rs consist of IL-4Rα and the common gamma chain cytokine receptor, whereas type II IL-4Rs are made of IL-4Rα and IL-13Rα1. IL-4 can signal via both type I and type II IL-4Rs, whereas IL-13 only signals via the type II IL-4R. Additionally, IL-13 can bind to IL-13Rα2, which is thought to be a non-signaling decoy receptor of very high affinity to IL-13. Currently, there are several treatment strategies in clinical use that interfere with IL-4R signaling. For targeting both IL-4 and IL-13 signaling Dupilumab, a monoclonal antibody blocking IL-4Rα, and Pitrakinra, an IL-4Rα antagonist, exist. For more selectively blocking of IL-13, Lebrikizumab, a monoclonal antibody targeting IL-13, is of use. Downstream of the IL-4R, the Janus kinases (JAKs) JAK1, JAK2, and JAK3 can the inhibited by the use of the small molecule JAK inhibitors Upadacitinib and Tofacitinib (see also section Biologics and Small Molecules Targeting the IL-4R Signaling Axis).

Upon the establishment of a type 2 cytokine environment a positive feed-back loop is initiated where more naïve T cells differentiate into T_H_2 cells and stimulation of eosinophils, basophils, and ILC2 takes place. B cells respond to type 2 cytokines by isotype switching to IgE and production of antibodies. Macrophages develop under the influence of type 2 cytokines into alternatively-activated macrophages (also termed M2 macrophages).

At barrier organs, such as the skin, lungs, and intestine, ILC2 are more numerous than in internal organs without barrier function. As ILC2 likely serve to amplify type 2 immune responses initiated by tissue and tissue-resident myeloid cells carrying PRRs, it is conceivable that type 2 immune responses at non-barrier sites differ from those at external and internal barriers. In this context, cobalt chromium microparticles injected intraperitoneally caused a type 2 inflammation dependent on IL-33 release by macrophages, followed by recruitment of neutrophils and production of IL-4, IL-5, IL-13, arginase-1, chitinase-like protein 3 (Chil3 or Ym1), and resistin-like molecule (RELM)-α (77).

In the lung, type 2 immune responses induce goblet cell hyperplasia. These cells produce mucins and anti-nematode protein RELM-β (78, 79). RELM-β together with RELM-α and arginase-1, produced by epithelial cells and fibroblasts, respectively, are also involved in tissue repair and deposition of extracellular matrix, which in the context of helminth infections can serve to encapsulate and trap the parasite. Both IgG and IgE antibodies, produced by B cells, help to limit the motion and fecundity of the worms (80). Antibodies are also crucial for surface labeling of pathogens, which favors opsonization and antibody-dependent cell-mediated cytotoxicity (ADCC). ADCC is mediated via Fc receptors expressed on many innate immune cells. Altogether, these Fc receptor-bearing cells contribute to the reduction of worm fitness, lower transmission, and expulsion, which all serve to protect the body from nematode infections.

Whether and how neutrophils are involved in type 2 immunity and which role the affected barrier vs. non-barrier tissue plays, remains unclear. In mouse models of helminth infection, neutrophils have been reported to have a beneficial role during the early pulmonary stages (15), however this also comes at the price of increased tissue damage (81, 82). Conversely, in human type 2 inflammatory disorders neutrophils have been reported to be absent (17). The role of neutrophils in type 2 immune responses will be further discussed later.

Uncontrolled type 2 immune responses can lead to the development of allergic diseases, including allergic conjunctivitis, allergic rhinitis, allergic asthma, allergic gastrointestinal disorders, and atopic dermatitis (AD). These diseases are characterized by elevated levels of type 2 cytokines and accumulation of the above-mentioned immune cells (83). Dupilumab, a monoclonal antibody blocking IL-4Rα (Figure 3), has been shown to be effective as a treatment of moderate-to-severe AD and moderate-to-severe asthma (84, 85). Thus, interference with this central type 2 cytokine receptor subunit can control some of these allergic diseases.

### Type 3 Immunity

Type 3 immunity is typically directed against extracellular bacterial and fungal infections, and it is characterized by the presence of the effector cytokines IL-17 and IL-22, which are prominently synthesized by type 3 ILCs (ILC3) and IL-17-producing T_H_ (T_H_17) cells (86). Moreover, IL-26, TNF, and granulocyte-macrophage colony-stimulating factor (GM-CSF) are also produced during type 3 immunity. Fibroblasts, epithelial cells, macrophages and, particularly, neutrophils become activated in response to these cytokines. IL-17 and GM-CSF induce extensive recruitment, activation and survival of neutrophils (87). IL-22 promotes epithelial cell homeostasis and has also been found to be important in antimicrobial defense (88).

Type 3 immunity is characterized by extensive neutrophil infiltration. Neutrophils are crucial for protection against and clearance of fungi (89). Also, immunity to certain encapsulated extracellular bacteria (e.g., *Staphylococcus aureus*) depends on efficient recruitment and activation of neutrophils. The importance of neutrophils in anti-fungal and anti-bacterial immunity is also apparent in subjects suffering from CGD and in patients receiving IL-17-targeting biologic agents (biologics), as these individuals are more susceptible to fungal and staphylococcal infections (90, 91).

A typical pathologic manifestation characterized by a type 3 immune response is the chronic-inflammatory skin disease psoriasis (92). Notably, neutrophil-rich microabscesses (termed Munro's microabscesses) in the uppermost layers of the epidermis are a characteristic hallmark of the skin lesions in plaque-type psoriasis. Inhibition of IL-17 by the use of IL-17-targeting biologics is very effective in psoriasis.

## Neutrophils in Type 2 Immune Responses

### The Importance of Neutrophils During Helminth Infections

Several groups have shown that neutrophils are important for limiting parasite survival and spreading in mouse models of helminth infections (15, 81, 82). Upon inoculation of mice with infective third-stage larvae of *Nippostrongylus brasiliensis*, the larvae migrate via the lungs to the intestine. Around the same time as the larvae arrive to the lungs, an increase of lung-infiltrating neutrophils is observed, which most likely represents a rapid mechanism to contain spreading. Thus, both mouse and human neutrophils have been shown to kill helminth larvae *in vitro*, when collaborating with macrophages (15). In fact, there are a number of studies showing that neutrophils and macrophages collaborate in immobilization and killing of these parasites. In different nematode infectious models, neutrophils and macrophages have been described to cluster together around the pathogen, however, neither of the two cell types was able to kill the larvae efficiently by itself (93–95).

As previously described, neutrophils are professional phagocytes, which is important in situations where the microbe is small. On the contrary, helminths are macropathogens and due to their size impossible for a neutrophil to ingest. Instead, neutrophils take advantage of other effector functions, namely degranulation and NET formation. Guided by virulence factors and chemokines neutrophils and other granulocytes arrive to the site of infection. Interaction between antibodies covering the helminth and Fc receptors on the cells causes ADCC-mediated degranulation (96). Complement factors serve a similar function as antibodies and can lead to complement-dependent cytotoxicity (15). Although eosinophils are acknowledged as the main effector granulocytes in helminth infestations, basophils and neutrophils are also recognized for their importance (97, 98). Both neutrophil granule proteins and NETs are efficient effector mechanisms to trap helminth larvae (99). Exposure of human and mouse neutrophils to *Strongyloides stercoralis* induces an even faster formation of NETs than the extremely potent NET stimulator phorbol 12-myristate 13-acetate (PMA).

Even though neutrophils have a very short life span, they are capable of initiating immune responses that persist long after they die. One example of this is priming of macrophages. M2 macrophages have been shown to rapidly surround nematodes and facilitate killing and clearance during helminth infection, particularly, during secondary infection (100). This priming of macrophages to become M2 macrophages during an initial infection was, however, only possible in the presence of neutrophils. Without neutrophils, macrophages could not efficiently adapt an M2-like transcriptional phenotype, which also resulted in impaired helminth expulsion.

All these studies support the importance of neutrophils especially in the early stages of a type 2 immune response against helminths. However, with activated neutrophils comes unavoidable tissue damage (82), and it is therefore important that activated neutrophils are kept in check.

### Type 2 Cytokines Dampen Neutrophil Functions

#### Evidence From Mouse Models

In contrast to the apparent importance of neutrophils in helminth infections, there is accumulating evidence that IL-4R signaling by IL-4 and IL-13 inhibits neutrophil effector functions (21). Woytschak et al. found that treatment of mice with IL-4 during cutaneous and systemic bacterial infections increased bacterial and disease burden, while blood neutrophil counts, neutrophil migration, and overall survival of animals decreased (19). The same trend was observed during sterile inflammation induced by using G-CSF, IL-1β, or monosodium urate crystals. Peripheral neutrophils of mice treated with IL-4 were found to have lower CXCR2 expression levels, which fits neatly with their impaired migratory ability. Conversely, bone marrow neutrophils of IL-4-injected animals expressed higher levels of CXCR4, offering an explanation for their decreased egress into the blood stream. All these effects were not observed in IL-4Rα-deficient mice as well as animals treated with a blocking anti-IL-4 monoclonal antibody. Moreover, mice lacking the IL-4Rα were shown to survive a systemic infection with *Listeria monocytogenes* that was lethal for wild-type control animals (19). Throughout all of the experiments performed in this work, the neutrophils behaved homogenously and there was no observation of duality that could hint at the presence of distinct *bona fide* neutrophil subpopulations.

Chen and colleagues found that neutrophil infiltration into the lungs upon infection of mice with the helminth *Nippostrongylus brasiliensis* was associated with IL-4R signaling (82). In this model, IL-4Rα-deficient mice presented with increased pulmonary neutrophil infiltration and worse disease scores, while neutrophil depletion led to a decrease of acute lung injury. In a mouse model of RA, a disease where neutrophils are believed to play a major role in the pathogenesis (101), Schmid et al. demonstrated that treatment of mice with IL-4 protected from joint inflammation (102). Other groups made similar observations treating RA with IL-4 and identified neutrophils and macrophages as the main targets of the therapy (103, 104). In a study combining helminth infection and RA, elicitation of a type 2 immune response by the parasite improved joint inflammation (105). In a recent publication, Harris and colleagues showed that IL-4R signaling under hypoxic conditions directly inhibited the survival of human neutrophils (106). These results highlight that IL-4R signaling during hypoxic acute respiratory distress syndrome, protects against neutrophil-induced lung injury. Under normoxic conditions, IL-4 or IL-13 did not appear to increase neutrophil apoptosis (20). Thus, IL-4R signaling seems to have yet another way of limiting neutrophil actions and protect against the harmful consequences of uncontrolled neutrophils.

As discussed earlier, micro- and nanoparticles are potent inducers of type 2 immunity. Since the body cannot degrade them, microparticle-elicited reactions often do not resolve, but lead to repeated waves of inflammation and immune cell influx. Here, IL-4R-mediated dampening of neutrophil responses again may serve as a preservation mechanism because neutrophils will not be able to clear the microparticles, but will only potentiate tissue damage while trying to do so.

Using a unique intravital imaging method, Wang et al. studied the migration behavior of neutrophils after a focal thermal injury in the liver. They found that after a short extensive influx, neutrophils proceeded to reenter the vasculature 12 h after injury and were finally recruited to the bone marrow in a CXCR4-dependent manner (107). Here, neutrophils were found to be responsible for the creation of paths through the tissue which facilitated vascular regrowth and access for other immune cells. Woytschak et al. found that treatment of neutrophils with IL-4 reduced their ability to migrate toward CXCL2 and resulted in downregulation of its receptor CXCR2, but not CXCR4 (19). Therefore, IL-4R engagement on neutrophils may not only dampen their migration toward CXCL2, but also promote reverse migration by shifting the balance toward signaling via CXCR4.

Ma and colleagues showed that the local phenotype of neutrophils change over time after myocardial infarction in mice (108). They found that proinflammatory N1 neutrophils dominated at day 1, whereas anti-inflammatory N2 neutrophils prevailed at days 5 and 7 after injury while they were not detected in peripheral blood. Tissue repair and fibrosis is part of the remodeling taking place after myocardial infarction. It has been shown that IL-4 and IL-13 are key drivers of these processes (109). It is therefore possible that the emerging tissue repair environment with type 2 cytokine milieu shifts the neutrophils from a pro-inflammatory N1 to an anti-inflammatory N2 phenotype. Although neutrophils seem to lose their pro-inflammatory phenotype in a type 2 immune milieu and may even contribute to the resolution of inflammation, they are distinct from conventional myeloid-derived suppressor cells (MDSCs) since the former are mature cells that change their phenotype in response to type 2 cytokines while the latter form newly from myeloid precursors in response to G-CSF/GM-CSF, IL-6, and a variety of other cytokines (110). There have, however, been reports of IL-4R signaling being important for the immunosuppressive nature of MDSCs (111, 112).

#### Translation to the Human Setting

Evidence of IL-4R signaling inhibiting neutrophils not only covers preclinical studies in mice, but there is also clinical data suggesting that this mechanism is evolutionarily conserved. Impellizzieri et al. recently demonstrated that incubation of human neutrophils with IL-4 or IL-13 significantly reduced their ability to migrate toward CXCL8 and produce NETs (20). These findings were mirrored when using freshly-isolated neutrophils from allergic patients with acute symptoms. Moreover, IL-4- or IL-13-treated neutrophils from healthy donors and neutrophils from allergic individuals showed lower surface expression of CXCR2 and its functional twin CXCR1, compared to untreated neutrophils from healthy donors, whereas CXCR4 expression was unaltered (20). The decrease in migratory ability *in vitro* upon IL-4 stimulation was confirmed and extended in a humanized mouse model, where an air pouch was induced in the back of NOD-*Prkdc*^*scid*^*-Il2rg*^*null*^ (NSG) mice, followed by triggering of local inflammation by injection of CXCL8 and lipopolysaccharide in the air pouch and intravenous adoptive transfer of human neutrophils. Flow cytometric analysis of the air pouch content revealed significantly lower counts of human neutrophils when the cells were pretreated with IL-4 as opposed to controls, indicating that IL-4R signaling in human neutrophils leads to impaired migration also *in vivo*. In accordance with the findings of Woytschak et al. with mouse neutrophils, Impellizzieri et al. did not find a duality in neutrophil functionality in their experiments that would hint at different *bona fide* subpopulations.

#### Biologics and Small Molecules Targeting the IL-4R Signaling Axis

In a small clinical trial with patients suffering from plaque-type psoriasis, an autoimmune disease characterized by type 3 inflammation and cutaneous neutrophil infiltration, treatment with IL-4 resulted in marked disease improvement (113); this therapeutic effect was likely due to a shift in the cytokine milieu from a type 3 to a type 2 immune response as well as a direct inhibition of neutrophils by IL-4. Conversely, in AD, an inflammatory skin disorder known for its type 2 cytokine signature, afflicted individuals often suffer from recurrent skin infections (18). Here, neutrophils are conspicuously absent in both healthy and lesional skin (17, 114). However, neutrophil chemoattractants were found to be elevated similarly in psoriatic and atopic skin (115), thus the stark difference in skin-infiltrating neutrophils in psoriasis and AD cannot be explained by differences in chemoattractants. Moreover, treatment of moderate-to-severe AD patients with the IL-4Rα-blocking antibody Dupilumab has been shown to not only significantly decrease disease burden, but also lower the incidence of skin infections (84, 116, 117). This protective effect against infections has been observed also for other type 2 immune diseases when treated with biologics targeting the IL-4R complex, such as the anti-IL-13 antibody Lebrikizumab in asthma and AD (118, 119) and the IL-4Rα-blocking agent Pitrakinra in asthma (120). There are also small molecule inhibitors of the IL-4R signaling axis targeting receptor-associated JAKs. Since JAKs are shared between different cytokine receptors, they are less selective for IL-4 or IL-13 signaling, but can also be used to suppress other pathways. Tofacitinib inhibits JAK1 and JAK3 and is approved for the treatment of RA and psoriatic arthritis (76). It has also been investigated for use in AD, both as systemic and topical treatment (121, 122). The selective JAK1 inhibitor Upadacitinib is currently being investigated as an alternative to tofacitinib for treating RA and psoriatic arthritis (76), but it may also be interesting for the treatment of type 2 immune diseases since IL-4Rα also uses JAK1 for signaling (Figure 3).

None of the studies involving IL-4 as a therapeutic agent or inhibitors of the IL-4/IL-13-signaling axis mentioned here, however, directly examined neutrophil activity before, after or during treatment. Therefore, we can only speculate that the amelioration of plaque-type psoriasis upon IL-4 treatment and the decrease in infections in type 2 diseases upon IL-4R signaling blockade is, at least in part, the direct result of increased or decreased IL-4R signaling on neutrophils, respectively. Further investigations focusing on neutrophils in these disease and treatment conditions will reveal to what extent direct IL-4R-mediated neutrophil inhibition contributes to the clinical pictures.

Taken together, the growing body of evidence indicates that IL-4 and IL-13 exert an inhibitory effect on neutrophils. Such inhibition affects neutrophil migration and tissue infiltration as well as neutrophil effector functions. These effects limit neutrophil-mediated tissue damage. Although some of these effects could be the result of the classical type 1 and type 3 vs. type 2 immune regulation, the aforementioned studies clearly prove that cell-autonomous IL-4R signaling directly curtails mouse and human neutrophil effector functions.

### Hypothesis of Kinetic Involvement and Exclusion of Neutrophils in Type 2 Immune Responses

**Figure 4:**
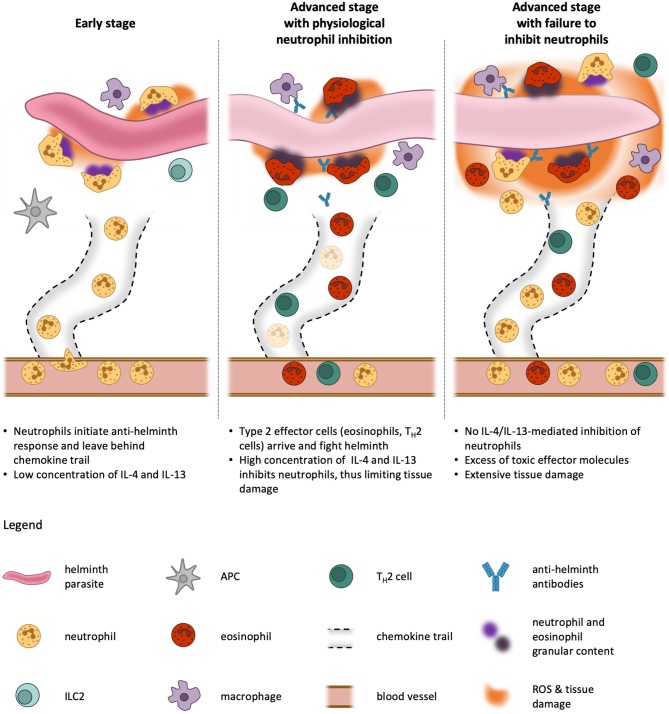
Kinetics of neutrophil activation and inhibition during type 2 immune responses. In an early phase of helminth infestation, neutrophils are quick to be recruited to the site of infection because they are abundant in the blood stream and are primed rapidly. On their way through the tissue, they leave behind channels and trails for other cells to migrate more efficiently toward the parasite. Once they encounter the helminth, the neutrophils release pro-inflammatory cytokines and exert effector functions against the invader. This facilitates the activation of neighboring antigen-presenting cells (APCs). At this stage, only little IL-4 and or IL-13 is produced by tissue-resident cells, such as type 2 innate lymphoid cells (ILC2), and thus neutrophil functions are unblunted **(Left)**. At later stages, APCs migrate to the lymph nodes where they initiate differentiation of T_H_2 cells, which in turn home to the infested tissue and produce their signature cytokines IL-4, IL-5, and IL-13. These cytokines cause recruitment and activation of professional type 2 effector cells, eosinophils and alternatively-activated macrophages, which use the preformed neutrophil channels to efficiently reach the pathogen. Our model proposes that, in a physiological condition, the presence of IL-4 and IL-13 dampens neutrophil activity and thus prevents excessive tissue damage, as these first responders are not needed anymore at this stage and would do more harm than good **(Middle)**. If this inhibitory mechanism fails, neutrophils continue to fight the helminth simultaneously with eosinophils and macrophages, resulting in profound tissue damage far beyond the necessary immune response to expulse the parasite **(Right)**. The size of the helminth parasite is not to scale, which would be larger in reality.

Having discussed the evidence suggesting an inhibitory role of IL-4R signaling on neutrophils, it seems awkward that neutrophils have been reported to play a significant role in type 2 immunity against helminth infections. Can these two seemingly contradictory paradigms fit together? We propose a hypothesis that involves a temporal separation of the two events and considers biological, physiological, and clinical aspects of type 2 immune diseases (Figure 4).

Neutrophils are abundant in the blood stream and very easily primed for attack of pathogens. Hence, as soon as a helminth parasite invades the body and is recognized as foreign, there are countless neutrophils already at the right place to extravasate and serve as a first line of host defense. By creating an inflammatory environment, neutrophils promote the activation of antigen-presenting cells (APCs). Moreover, pathogens killed by neutrophils in these very early stages may be easier for APCs to take up, digest, and present. Furthermore, starting from the blood vessel, neutrophils create a channel decorated with chemokine trails in the tissue, thus facilitating access for other immune cells (123). At this stage, neutrophils constitute the overwhelming majority of leukocytes in the tissue and there are only low concentrations of IL-4 or IL-13 produced by resident immune cells. Thus, the neutrophils are not inhibited. APCs then migrate to the lymph nodes where they initiate differentiation of antigen-specific naïve T cells to T_H_2 cells. These in turn home to the infected and inflamed tissue where they produce their signature type 2 cytokines IL-4 and IL-13. Due to clonal expansion, there soon are many activated T_H_2 cells present and the resulting cytokine milieu leads to inhibition of neutrophils, but also the recruitment and activation of eosinophils which are now taking over the neutrophils' job. Eosinophils are better equipped for fighting parasites and helminths, but take longer to be activated and accumulate in sufficient numbers. Simultaneous action of neutrophils and eosinophils would be detrimental for the surrounding host tissues. Thus, as soon as the type 2 immune response is established, the neutrophils step back and become quiescent due to IL-4R signaling.

In summary, the neutrophils bridge the time between pathogen invasion and the arrival of sufficient numbers of type 2 effector cells by initiating defense and help recruiting other immune cells. In doing so, they also facilitate the establishment of an inflammatory microenvironment. Since overshooting neutrophil activity would be destructive for host tissues, neutrophils are inhibited once they are not needed anymore. We thus postulate that the connection between IL-4R signaling and neutrophil inhibition has evolved as a safety mechanism to protect the body from neutrophil-inflicted harm.

## Author Contributions

CE, LH, and OB set the outline of the manuscript. CE and LH prepared the figures and wrote the first draft of manuscript with input from DI. OB revised and edited the manuscript and figures.

### Conflict of Interest

The authors declare that the research was conducted in the absence of any commercial or financial relationships that could be construed as a potential conflict of interest.
